# Local condiments from fermented tropical legume seeds modulate activities of critical enzymes relevant to cardiovascular diseases and endothelial function

**DOI:** 10.1002/fsn3.582

**Published:** 2018-02-08

**Authors:** Adedayo O. Ademiluyi

**Affiliations:** ^1^ Department of Biochemistry Federal University of Technology Akure Nigeria

**Keywords:** African locust bean, cardiovascular disease, fermentation, functional foods, soybean

## Abstract

Investigation into modulatory effects of local condiments produced from fermented legume (African locust bean and soybean) seeds on activities of enzymes relevant to endothelial function and cardiovascular disease (arginase, phosphodiesterase‐5, acetylcholinesterase, and, ecto 5′‐nucleotidase) in vitro was the focus of this study. The condiments were prepared according to traditional methods of fermentation. Thereafter, modulatory effects of aqueous extracts from the condiments on activities of the enzymes were subsequently carried out. Results showed the extracts significantly inhibited activities of arginase, phosphodiesterase‐5 and acetylcholinesterase, while the activity of ecto 5′‐nucleotidase was stimulated at sample concentrations tested. Thus, the observed enzyme modulatory properties exhibited by the condiments could be novel mechanisms to support their use as functional foods and nutraceuticals for the management of cardiovascular disease and associated endothelial dysfunction.

## 1 INTRODUCTION

Despite worldwide medical campaign, cardiovascular disease (CVD) remains the principal cause of death in both developed and developing countries. In 2008, the World Health Organization (WHO) estimated that 12.6% of all global deaths were caused by ischemic heart disease (WHO, 2013). By 2015, 17.7 million CVD‐related deaths were reported, amounting to 31% of all deaths globally; CVD now ranked number one global killer (WHO fact sheet, 2017). The more alarming fact is that more than 75% of all cases of CVD‐related deaths occur in low‐ and middle‐income countries (WHO fact sheet, 2017), with over 1 million cases (accounting for 5.5% of all global CVD‐related deaths) recorded in 2013 in sub‐Saharan Africa (Keates, Mocumbi, Ntsekhe, Sliwa, & Stewart, 2017). Futhermore, the fact that CVD is progressive and often with associated comorbidities makes this condition to be associated with significant socioeconomic implications, often requiring years of therapy and extensive health care costs (Hennekens, 1998). In 2011, CVD accounts for more than 275 billion dollars in the United States alone and is projected to go as high as over 500 billion dollars in direct hospital cost alone by the year 2030 (Mozaffarian et al., 2016).

The vascular endothelium is essential for vascular homeostasis by modulating vascular tone and inflammation responses. However, endothelial dysfunction is observed early in the pathogenesis of CVD and hence, a vital therapeutic target in several CVD, including hypertension, atherosclerosis, and vascular complications in diabetes mellitus (Brunner et al., 2005). While it is well known that the etiology of endothelial dysfunction is multifactorial, one of the major risk factor is considered to be impaired bioavailability of the vasodilator‐nitric oxide (NO) (Munzel, Heitzer, & Harrison, 1997). In fact, Kukreja et al. (2004) defines endothelial dysfunction as a reduction in bioavailability of NO. Hence, therapeutic strategies for ameliorating endothelial dysfunction majorly through increasing NO bioavailability are considered a positive step in the management of CVD. In lieu of this, modulations of activities of some enzymes (acetylcholinesterase, arginase, phosphodiesterase‐5 and ecto‐5′‐nucleosidase) has been studied as therapeutic targets in improving NO bioavailability, thereby ameliorating endothelial dysfunction and hence, CVD. Ecto‐5′‐nucleosidase hydrolyses ATP and AMP, respectively, to adenosine which serves as a vasodilator (Rådegran & Calbet, 2001). Phosphodiesterase is responsible for the hydrolysis of cyclic 3, 5 adenosine monophosphate (cAMP) and 3, 5 cyclic guanosine monophosphate (cGMP). Inhibitors of this enzyme function to conserve cGMP which is involved in NO‐mediated cGMP vasodilation (Kukreja et al., 2004). Nitric oxide synthase (NOS) is the enzyme involved in the synthesis of NO but shares a common substrate (l‐arginine) with the enzyme arginase (Durante, Johnson, & Johnson, 2007). An upregulated arginase activity makes l‐arginine less available for NO synthesis via NOS. Therefore, conserving bioavailability of NO could also be achieved through arginase inhibitors. Acetylcholine, like adenosine also serves as a vasodilator in the cardiovascular system (Shryock & Belardinelli, 1997) hence, their continual breakdown as a result of increased activity of acetylcholinesterase could be implicated in pathogenesis of many cardiovascular diseases as well as hypertension. Interestingly, natural product modulators of these enzymes with cardioprotective properties have been reported (Akinyemi et al., 2016; Girard‐Thernier, Pham, & Demougeot, 2015; Leal et al., 2016; Rahimi, Ghiasi, Azimi, Fakhari, & Abdollahi, 2010; Zhai et al., 2009).

Dietary intervention has been one major action plan advocated for the management of CVD (World Health Organization, 2013). Inclusion of legumes in dietary regimen for the management of CVD have been well advocated (Eckel et al., 2014; Widmer, Flammer, Lerman, & Lerman, 2015). Furthermore, legumes have gained remarkable attention in developing countries because of the continuous high demand for nutritious foods. Legumes are high in proteins, carbohydrates, and dietary fibers and are also endowed with other nutritional phytochemicals (Messina, 1999). Local condiments are produced from underutilized legumes through natural fermentation, and have traditionally served as important ingredients for preparing several foods in sub‐Saharan Africa (Ademiluyi, Oboh, Boligon, & Athayde, 2015). The medicinal properties of many fermented legume seeds have been associated with their rich content of phenolic phytochemicals (Ademiluyi, Oboh, Boligon, & Athayde, 2014; Ademiluyi et al., 2015). In addition, it has been studied that fermentation increases the phenolic contents of legume seeds (Oboh, Ademiluyi, & Akindahunsi, 2009). While the medicinal properties of fermented legume seeds including fermented pigeon pea, Bambara groundnut, African locust bean, and soybean in the management of CVD such as hypertension has been reported (Ademiluyi & Oboh, 2015; Lee, Lai, & Wu, 2015), the possible underlying mechanisms have not been sufficiently studied. Therefore, this study was designed to investigate the modulatory effect of extracts from fermented locust bean and soybean on activities of AChE, PDE‐5′, arginase, and ecto‐5′nucleosidase enzymes which are linked to endothelial function and CVD.

## 2 MATERIALS AND METHODS

### 2.1 Chemical and reagents

Para‐nitrophenylphenylphosphonate (PNPPP), acetylthiocholine iodide, 5,5′‐dithiobio‐(2‐nitrobenzoic acid) (DTNB), dimethyl aminobenzaldehyde, and adenosine monophosphate were obtained from Sigma–Aldrich Co. (Steinheim, Germany). All other chemicals used were of analytical grade and glass distilled water was used throughout.

### 2.2 Samples collection

African Locust bean (*Parkia biglobosa*) and soybean (*Glycine max* (L). Merrill) seeds were collected from Akintade village, a suburb of the Federal University of Technology, Akure, Nigeria. Authentication of the seeds was carried out at the department of Crop, Soil, and Pest Management, Federal University of Technology, Akure, Nigeria.

### 2.3 Preparation of the fermented legume condiments

The fermented legume condiments were prepared as previously reported by Ademiluyi et al. (2015). This involves soaking the seeds in warm water for 1 hr and dehulled by rubbing between palms. Subsequently, the seeds were rinsed under running tap water and cooked for about 3 hr until adequately softened. Thereafter, the cooked seeds were spread in baskets covered with clean banana leaves and kept in a jute sack. This was subjected to wild fermentation for 3 days at 37°C to produce the local condiment. The condiments produced were oven‐dried at 45°C, milled into powder and stored in refrigerator prior to extraction process.

### 2.4 Extraction process

The dried fermented condiments were defatted with diethyl ether (2:5 w/v) for 12 hr and centrifuged at 2415 × *g* for 20 min at 4°C, before the organic layer was removed. Thereafter, the samples were extracted with water (1:10 w/v), centrifuged at 1677 × *g* for 20 min while the supernatant was collected and filtered (Whatmann filter paper). The filtrate was lyophilized and subsequently reconstituted with water (10 mg/mL) for further analysis.

### 2.5 Preparation of cardiac tissue homogenate

Albino rats weighing 150–200 g were used for this study. Rats were maintained at 25°C, on a 12 hr light/12 hr dark cycle. They were acclimatized under these conditions for 2 weeks before the experiment. The rats were allowed free access to food and water ad libitum. The handling and use of the animals were in accordance with NIH Guide for the care and use of laboratory animals. The rats were immobilized by cervical dislocation and rapidly dissected. Heart tissue was removed and homogenized in cold (0.1 mol/L) phosphate buffer (pH 7.4). The supernatant which was used as source of the enzyme was prepared by centrifuging the homogenate for 20 mins at 1073 × *g* in a Kenxin refrigerated centrifuge Model KX3400C. (KX3400C, KENXIN Intl. Co., Hong Kong).

### 2.6 Enzyme assays

#### 2.6.1 Arginase assay

Arginase assay was performed according to previously reported method (Oboh, Adebayo, Ademosun, & Boligon, 2017). In brief, a solution containing crude enzyme and various concentration of extracts were preincubated for 5 min in 0.01 mmol/L Tris‐HCl buffer (pH 7.5) containing 0.05 mmol/L MnCl_2_. The reaction was initiated by addition of 50 mmol/L l‐arginine followed by incubation at 37°C for 10 min. The reaction was terminated by adding Ehlrich's reagent (p‐dimethylaminobenzaldehyde in 3.6 N HCl). The amount of urea produced was measured spectrophotometrically at 450 nm and arginase inhibition was subsequently calculated. The control experiment was performed without the test extracts and the arginase activity/inhibition was expressed as percentage of control.

#### 2.6.2 Ecto‐5′ nucleotidase assay

Ecto‐5‐nucleotidase assay was determined as previously reported (Ademiluyi, Ogunsuyi, & Oboh, 2016). The reaction mixture consisted of 10 mmol/L MgSO4 and 100 mmol/L Tris–HCl buffer, pH 7.5. 20 μl of enzyme preparation was added to the reaction mixture and preincubated at 37°C for 10 min. The reaction was initiated by the addition of AMP to a final concentration of 2.0 mmol/L and proceeded for 20 min. In all cases, the reaction was stopped by the addition of 200 μl of 10% trichloroacetic acid (TCA). The tubes were chilled on ice for 10 min and the released inorganic phosphate (Pi) was assayed by the method of Chan, Delfert, and Junger (1986), using KH_2_PO_4_ as standard. The ecto‐5′‐nucleotidase activity/inhibition was expressed as percentage of control.

#### 2.6.3 Acetylcholinesterase assay

Acetylcholinesterase (AChE) assay was assessed by a modified colorimetric method (Perry, Houghton, Theobald, Jenner, & Perry, 2000). This was carried out in a reaction mixture containing 200 μl of homogenate, AChE solution in 0.1 mol/L phosphate buffer pH 8.0, 100 μl of a solution of 5,5′‐dithio‐bis(2‐nitrobenzoic) acid (DTNB 3.3 mmol/L), dilutions of the extracts, and 500 μl of 0.1 mol/L phosphate buffer, pH 8.0. After incubation for 20 min at 25°C, 100 μl of 0.05 mmol/L acetylthiocholine iodide was added as the substrate, and AChE activity was determined from the absorbance changes at 412 nm for 3 min, using spectrophotometer. The AChE activity/inhibition was expressed as percentage of control.

#### 2.6.4 Phosphodiesterase‐5 inhibition assay

The phosphodiesterase‐5 assay was determined as previously reported (Oboh, Ademiluyi, Oyeleye, Olasehinde, & Boligon, 2017). Briefly, solution containing the crude enzyme with various dilutions of extracts in 20 mmol/L Tris‐HCl buffer (pH 8.0) containing 1 mmol/L CaCl_2_ and 50 mmol/L NaCl was incubated at 37°C for 10 min. Thereafter, 200 μl of substrate (5 mmol/L p‐nitrophenyl phenylphosphonate) was added. The intensity of p‐nitrophenol produced was measured spectrophotometrically at 405 nm. The control experiment was carried out without addition of extract. Phosphodiestrease‐5 modulatory property of the extract was subsequently calculated and expressed as percentage of control.

### 2.7 Data analysis

Results were expressed as mean ± SD. Mean values were appropriately analyzed and compared, using two way analysis of variance (ANOVA) followed by Bonferroni post hoc test; significance was accepted at *p* < .05; *p* < .01; *p* < .001. All statistical analysis was carried out, using GraphPad Prism version 5.00 for Windows.

## 3 RESULT

**Figure 1:**
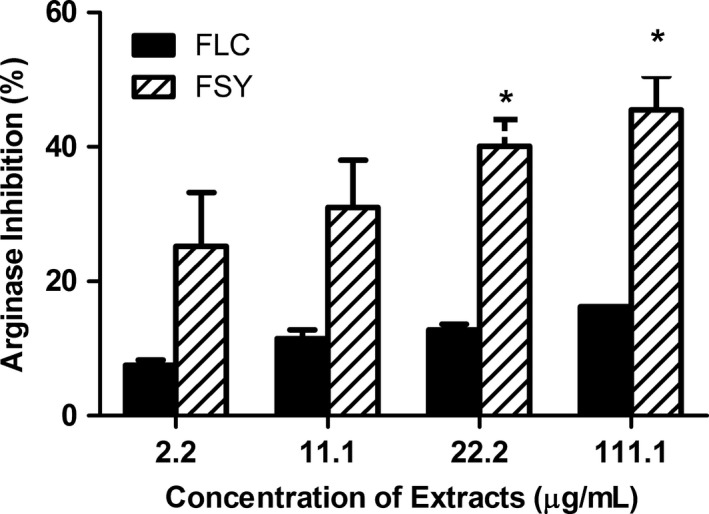
Arginase Inhibitory Effect (%) by Fermented Locust Bean and Fermented Soybean Extracts. Values represent mean ± *SD*. Values with different letters are significantly different at * *p* < .05

The interaction of the fermented locust bean (FLC) and fermented soybean (FSY) extracts with arginase activity is presented in Figure 1 and the result showed that both extracts inhibited arginase activity in concentration‐dependent manner (2.2–111.1 μg/ml). According to the Two‐way analysis of variance followed by Bonferoni posttests for significance level of means, we observed that 22.1 and 111.1 μg/ml FSY extract exhibited a significantly higher (*p* < .05) arginase inhibitory effect than FLC extract.

**Figure 2:**
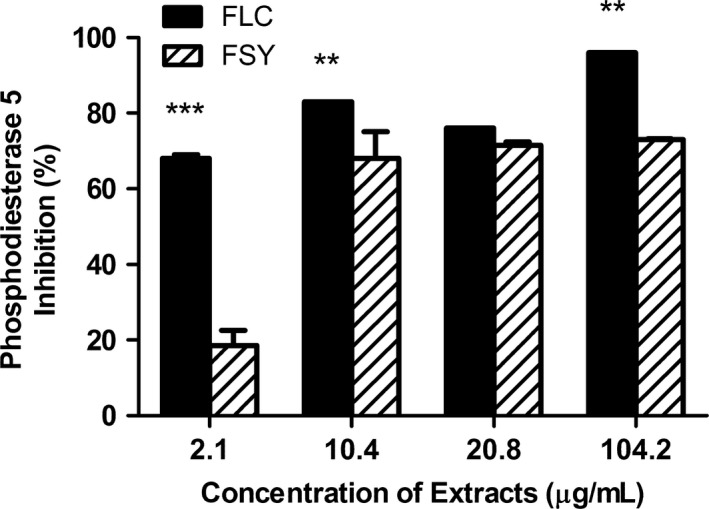
Phosphodiesterase‐5 Inhibitory Effect (%) by Fermented Locust Bean and Fermented Soybean Extracts. Values represent mean ± *SD*. Values with different letters are significantly different at **p < .01; ***p < .001

In Figure 2, this study revealed that both FLC and FSY extracts exhibited phosphodiesterase‐5 (PDE‐5) inhibitory effects at all concentrations tested (2.1–104.2 μg/ml). However, results from the Two‐way analysis of variance followed by Bonferoni posttests for statistical significance revealed that at 2.1 μg/ml, 10.4 μg/ml and 104.2 μg/ml, FLC extract exhibited significantly higher (*p* < .01; *p* < .001) PDE‐5 inhibitory effect compared to the FSY extract.

**Figure 3:**
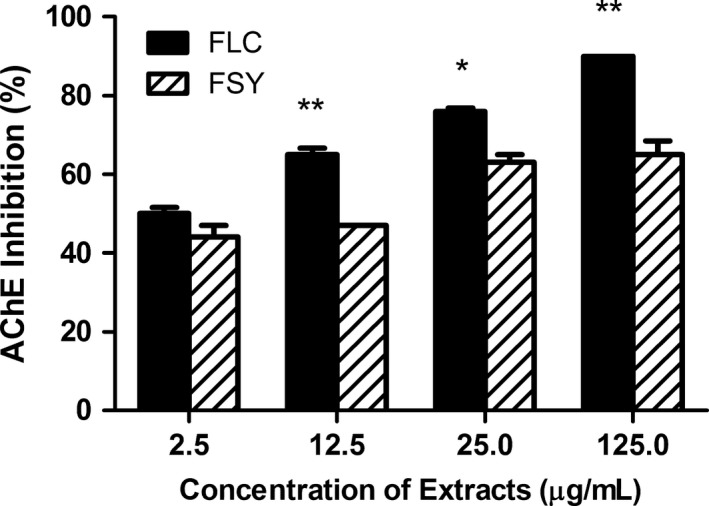
Acetylcholinesterase Inhibitory Effect (%) by Fermented Locust Bean and Fermented Soybean Extracts. Values represent mean ± *SD*. Values with different letters are significantly different at * *p* < .05; ** *p* < .01

Also, the acetylcholinesterase (AChE) inhibitory effect of both extracts were also studied and the result presented in (Figure 3). The result revealed that both extracts inhibited acetylcholinesterase activity in a concentrated‐depended manner (2.5–125 μg/ml). There was also significant difference in the inhibitory effects, with FLC extract producing a significantly higher (*p* < .05; *p* < .01) AChE inhibitory effect than FSY extract at all but the lowest concentration tested.

**Figure 4:**
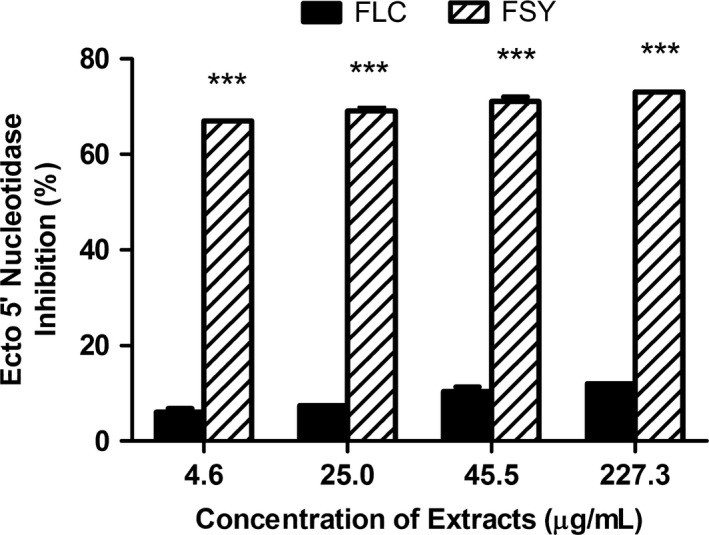
Ecto‐5‐nucleotidase Inhibitory Effect (%) by Fermented Locust Bean and Fermented Soybean Extracts. Values represent mean ± *SD*. Values with different letters are significantly different at ***p < .001

Lastly, the effect of FLC and FSY extracts on Ecto‐5′nucleosidase (E5NT) activity was investigated (Figure 4). This showed that the extracts stimulated the activity of E5NT concentration dependently (4.6–227.3 μg/ml), with FSY extract producing a significantly higher (*p* < .001) stimulatory effect than FLC extract at all concentrations tested when results from the Two‐way analysis of variance followed by Bonferoni posttests for statistical significance was considered.

## 4 DISCUSSION

This study revealed that both fermented locust bean (FLC) and fermented soybean (FSY) extracts inhibited the activity of arginase in cardiac tissue homogenate. Elevated arginase activity has been identified in cardiovascular diseases such as hypertension. (Morris et al., 2003). Cardiovascular disease (CVD) is often characterized by endothelial dysfunction and decreased bioavailability of the vasodilator‐nitric oxide (NO). Therefore, in conditions of CVD, inhibition of arginase activity is a critical mechanism by which proper endothelial function can be restored via increased bioavailability of NO. NO is synthesized by nitric oxide synthase (NOS) from l‐arginine. However, l‐arginine is a common substrate for both NOS and arginase which functions to hydrolyze l‐arginine to urea and l‐ornithine (Durante et al., 2007). Consequently, inhibition of arginase activity has been suggested as a practical therapeutic means of increasing l‐arginine as a substrate for NOS, and ensuring bioavailability of NO. Hence, the observed arginase inhibitory effects of both samples in this study suggest their potential role in the management of CVD by ensuring availability of l‐arginine for NO synthesis.

Another mechanism by which the vasodilator role of NO can be conserved is through PDE‐5 inhibition. In this study, the extracts were able to inhibit the activity of PDE‐5 in cardiac tissue homogenate. This could also help explain in part, the underlying mechanism behind the therapeutic potential of these condiments for the prevention and management of CVD. This is in agreement with earlier reports on the PDE‐5 inhibitory properties of different plant extracts used in folkore medicine for management of CVD (Lokesh & Amitsankar, 2012; Rahimi et al., 2010). Although PDE‐5 inhibitors are primarily used for therapeutic management of erectile dysfunction (Porst et al., 2001), a growing body of evidence are supporting the cardioprotective role of PDE‐5 inhibitors. PDE‐5 inhibitors such as sildenafil functions by inhibiting PDE‐5, a ubiquitous enzyme that hydrolysis cyclic 3,5 cyclic guanosine monophosphate (cGMP), thus accumulating cGMP for its NO‐mediated cGMP vasodilator role (Kukreja et al., 2004). Hence, some of the proposed mechanisms of cardioprotective properties of PDE‐5 inhibitors are generation of NO and accumulation of cGMP in the myocardium through inducible and endothelial nitric‐oxide synthase (iNOS and eNOS) (Das, Xi, & Kukreja, 2005).

Apart from NO, another vasodilator of physiological and therapeutic importance is adenosine. Adenosine is a ubiquitous extracellular signaling molecule which mediates several important physiological processes; it induces vascular dilation, performs modulatory roles in the sympathetic nervous system, exhibits antithrombotic properties, and elicits blood pressure and heart rate reduction (Buckert, Dewes, Walcher, Rottbauer, & Bernhardt, 2013). Reports have it that endogenous administration of adenosine offers cardioprotective properties via adenosine receptor activations (Shryock & Belardinelli, 1997). Therefore, maintenance of high micromolar endogenous concentration of adenosine is of cardioprotective benefits (Shryock & Belardinelli, 1997). Ecto‐5′nucleosidase (E5NT) is the major enzyme responsible for phosphohydrolysis of 5′‐adenosine monophosphate to produce adenosine, and it is widely expressed in endothelial cells. This study revealed that both fermented legume seed extracts stimulate the activity of ecto‐5′nucleosidase in rat cardiac tissue homogenate. Through this mechanism, the extracts could stimulate increased production of adenosine which could help in maintaining vascular tone and ensure vasodilation.

The extracts also inhibited acetylcholinesterase (AChE) activity in cardiac tissue homogenate. The role of AChE in heart has been widely studied (Ezz, Khadrawy, & Mourad, 2015; Katz, Schwarz, Yuen, & LeJemtel, 1993; Roy, Guatimosim, Prado, Gros, & Prado, 2014). AChE catalyzes the conversion of the neurotransmitter acetylcholine to choline. However, acetylcholine, like adenosine also serve as a vasodilator in the cardiovascular system (Shryock & Belardinelli, 1997) and hence, their continual breakdown as a result of increased activity of AChE could be implicated in pathogenesis of many cardiovascular diseases as well as hypertension. The dilator role of acetylcholine in coronary blood vessels has been shown to be mediated by nitric oxide (Lefroy, Crake, Uren, Davies, & Maseri, 1993). Therefore, inhibition of AChE activity by the extracts in rat cardiac tissue homogenate could induce the bioavailability of acetylcholine which produces vasodilator effects that can help maintain vascular blood flow. This could also contribute significantly to the therapeutic potentials of these local condiments in the prevention and management of cardiovascular diseases.

The observed bioactivities of the condiments understudied could be attributed to their constituent phytochemicals. Both fermented locust bean and soybean seeds have been reported to be rich in several bioactive phytochemicals, especially polyphenols. Ademiluyi and Oboh (2012) reported both seeds to be rich in phenolic phytochemicals with as high as 169.46 mg/100 g total phenol content reported for FSY, which was 23% higher than the value reported for FLC. These reports also agree with Oluwaniyi and Bazambo (2014), and Romero, Doval, Sturla, and Judis (2004) who also reported an abundance of total phenol contents in fermented locust bean and soybean seeds, respectively. Earlier reports (Ademiluyi et al., 2014, 2015) that characterized the polyphenolic contents of both FLC and FSY via HPLC‐DAD reported the abundance of gallic acid, caffeic acid, quercetin, naringin, and kaempferol in both samples. Copious research findings have reported the bioactive properties of polyphenols and polyphenol‐rich plant extracts to include arginase (Girard‐Thernier et al., 2015; Oboh et al., 2017); PDE‐5 (Oboh, Ademiluyi, et al., 2017; Rahimi et al., 2010); AChE (Ademiluyi et al., 2015; Go et al., 2016; Mathew & Subramanian, 2014) and ecto‐5′nucleosidase (Akinyemi et al., 2016; Leal et al., 2016; Oboh, Adewuni, Ademosun, & Olasehinde, 2016) modulatory effects. Therefore, the observed enzyme modulatory effects of the condiments extract in this study could be associated with their abundance polyphenolic contents as previously reported.

In conclusion, this study has been able to show that extracts from local condiments produced from fermented locust bean and soybean significantly modulate the activities of enzymes (phosphodiesterase‐5, arginase, ecto 5‐nucleotidase, and acetylcholinesterase) relevant to endothelial function and cardiovascular disease in vitro in rat cardiac tissue homogenates. These enzyme modulatory properties could help explain possible new underlying mechanisms to support the use of these condiments as dietary intervention for the management of endothelial dysfunction and cardiovascular diseases.

## CONFLICT OF INTEREST

None declared.
